# Report of the Sanitary Commission on the Epidemic Yellow Fever of 1853

**Published:** 1855-07

**Authors:** 


### BOOK NOTICES.

Report of the Sanitary Commission on the Epidemic Yellow Fever of 1853, Published by Authority of the City Council of New Orleans.

The fearful epidemic of 1853 throughout the South, and especially in the ill-fated city of New Orleans. is still fresh in the minds of our readers. Its victims, many of them at least, were closely endeared to Northern hearts and Northern homes—too much so indeed to be soon forgotten.

During the summer, while the epidemic was raging with unparallelled violence, invading the most salubrious localities. and selecting its subjects from all classes of society, the attention of the city was thoroughly aroused to its sanitary condition. The Board of Health appointed the Hon. A. D. Crossman Mayor of the city, Drs. E. H. Barton, A. F. Axson, S. D. McNeil, J. C. Simonds and J. L. Riddell, to constitute a Commission, with instructions—

"1st. To inquire into the origin and mode of transmission or propagation of the late epidemic yellow fever.

2d. To inquire into the subject of sewerage and common drains, their adaptability to the situation of the city, and their influence on health.

3d. To inquire into the subject of quarantine, its uses and applicability here, and its influence in protecting the city from epidemics and contagious maladies, and

4th. To make a thorough examination into the sanitary condition of the city, into all causes influencing it in present and previous years, and to suggest the requisite sanitary measures to remove or prevent them, and into the causes of yellow fever in ports and other localities having intercourse with New Orleans."

From a carefully kept meteorological table, the following propositions are, in the opinion of the Commission, demonstrated:

1. What are the several meteorological conditions of yellow fever and cholera at the commencement, maximum intensity and declination of these two diseases when existing in their epidemic grades.

2. In comparison, it shows that cholera exists in a greater range of temperature and humidity than yellow fever.

3. That these diversities constitute the pabulum for its support, so far as the mere climatic condition is concerned.

4 That a higher solar radiation and atmospheric pressure exists during yellow fever periods than during cholera. Although the atmospheric pressure under which these two diseases prevail are shown by this average table to be about the same, the barometer continuing at a permanently higher grade, more regularly and constantly in yellow fever than in cholera, yet in this latter the fluctuations are much greater; indeed, it is so under all its climatic relations, as is abundantly shown in the large detailed table, too extensive for this summary, of which this is a very condensed abstract.

5. That for the existence of yellow fever a higher range of temperature and of dew point for its commencement and maximum intensity, and that a declension of the former (temp.) to less than 70°, and the latter (dew point) to near 60°, puts a speedy end to its *epidemic* existence.

6. That a larger quantity of rain usually falls, on an average, during the existence of yellow fever than during cholera.

7. The "drying power" is more variable during cholera than during yellow fever.

8. The average duration of epidemic yellow fever has been 58 33 days, and the period of its influence decreasing, while the average duration of cholera has been 37.66 days, and the period increasing.

The inquiry into the origin and mode of transmission or propagation of the epidemic was committed to Drs. Axson and McNeil.

The conclusions to which they have arrived, are the following :

That it has not been derived from abroad, but is of spontaneous origin :

That there existed here, as attested by our records, very peculiar meteorological conditions, known, by general experience, to be capable of producing, in co-operation with local causes, fatal and malignant forms of fever :

That these conditions were present in an exaggerated degree, and impressed upon the prevalent type of disease susceptibilities and habits, assimilating it to another and distinct form of fever :

That this showed in all those localities within the range of the meteorological state or influence, an infectiousness not necessary to, or characteristic of the fever, but purely casual and incidental, the iesult of physical causes, and which it loses as soon as those causes are changed or disappear.

The second inquiry was committed to Dr. Riddell. This portion of the report is of very great local interest, but would interest but few of our readers, except those living in our larger cities. From our knowledge of the city of New Orleans, we are satisfied that the plan recommended by Prof. R., if carried out, would do much towards securing for the city at least a partial exemption from the epidemics which have so often swept over it, and which have carried away so many of its most active and enterprising citizens.

Dr. Simonds was charged with the third inquiry, embracing the subject of quarantine. We think a more careful analysis of the facts presented by the correspondence should have been made in reference to this subject. The writer of this portion of tho report seems to believe in the contagiousness of yellow fever, although his language is rather indefinite—perhaps we should say cautious. He holds the following language, however, in reference ot the propriety of quarantine:

As regards the propriety of quarantine, the Commission are unanimously of the opinion that a quarantine should be established. Even if of no use as to yellow fever, it will serve to keep out other diseases, equally, if not more, deleterious to our city and its inhabitants. It may prevent the introduction of diseases known and acknowledged by all to be contagious or infectious. It will prevent the introduction of moribund emigrants, and thus by diminishing the number of interments in our city, enable us to ascertain more certainly its degree of salubrity—which cannot be done at present, on account of the disturbing influence thereof. It will require all vessels to arrive in a cleanly condition, and consequently, by compelling the officers to keep their vessels clean during the whole voyage, extend its beneficial effects for weeks anterior to their arrival, and therefore, conduce to the comfort and salubrity of those passengers whose circumstances are such that they cannot protect themselves nor enforce those requirements which are necessary.

The section of the report by Dr. Edward H. Barton occupies the larger portion of the volume. We have not time to enter into an analysis of it, and if we had we do not know that we could give as correct an idea of its contents as will be gathered from the following resume :

1st. That the insalubrity of New Orleans, which has now continued for so many years—with some remarkable exceptions—is not natural to her, or *necessarily* incidental to her position ; that it is the cause of the high price of everything, and the direct means of retarding her progress to prosperity, and which will continue to exist until effective measures are taken to remove it.

2d. That the direct and *inevitable* change of *climate alone*, is not the sole cause of the mortality of immigrants, but the union of the climatic with the terrene conditions under *different circumstances*, were the efficient agents in the destructive influence on each class of people as pointed out according to nativity ; that man cannot become acclimated to the second cause, or terrene (filth, etc.) anywhere, and that the acclimation to our first cause (or atmospheric) would be trifling, if the conditions constituting it were so modified, as was clearly shown to be, in our power.

3d. We have endeavored to prove what were the CONSTITUENTS of the epidemic yellow fever of 1853, that they consisted of certain atmospheric and terrene combinations; that these causes, so far as we had the means of ascertaining, were confined to the limits of the fever district; that it began with these causes and ended with them, throughout the limits of the epidemic region, and that when these ceased, so terminated its influence on man.

4th. That one of these causes (the atmospheric) is more or less present here every summer, and that when the second (or terrene) exists in sufficient amount, an *epidemic* is the certain result, so far as near sixty years' experience will go to prove it; that this terrene condition is mainly composed of extensive disturbances, or upturning or exposure of the original soil of the country; that without this there has been no such epidemic, although between the occurrence of some of them long periods have elapsed ; and that its ravages or malignity appears to have been pretty much in proportion to the extent of that disturbance.

5th. That for the existence of an *epidemic*, a wide pervading atmospheric cause being one of the essential elements, an *epidemic disease cannot be imported*, and that as a contagious disease cannot depend upon a *general cause* for its existence, but must derive its qualities from a specific one ; epidemic yellow fever is consequently not a contagious disease.

6th. That to constitute an *endemic yellow fever*, the difference of which from an epidemic was fully pointed out, that the *apparent contagion* was only the extension of the epidemic principle, a lesser degree of the same, or what was believed to be equivalent,

(filth of all kinds, and decomposing materials) with a lesser degree or intensity of the first or atmospheric constituent, were essential.

7th. That when these causes did not exist in a sufficiently high degree to produce yellow fever, intermittent, remittent, bilious, or other periodic fevers were the result, demonstrating by the clearest analogy that they proceeded from the same cause, and that they differed only in degree and intensity, a major amount of the very same materials being required to produce yellow fever, as a minor one does for bilious or periodic fevers.

8th. That all these fevers are produced from *local causes*, more or less extensive, and that the fevers, the result of these, were limited to these bounds, that these causes are well understood, and were extensively pointed out in detail, and wherever the epidemic *extended*, there were *causes to localize it*, that where these did not exist, the cases of the epidemic conveyed there did not extend, and that consequently, that all these fevers arising from bad air, are no more contagious or infectious the one than the other, the liability to them is limited to the bad or infected air and personal susceptibility; and finally, that these are *of the greatest importance in their practical bearing on sanitary measures.* And,

9th. That the temporary epidemic cholera, which occurred here early in December, it was shown, depended also upon two conditions, an atmospheric and a terrene; that the first of these was different from that required to produce epidemic yellow fever, although the second was believed to be the same.

From all which the following corollaries were deduced; viz:

1st. That an epidemic yellow fever in New Orleans, if produced by the causes stated in our third proposition, as believed, being known, is controlable, that is, *preventable.*

2d. That an endemic yellow fever, arising from the same or equivalent causes, as above, although in a lesser degree, can also be mainly, if not entirely controlled.

3d. That the causes of bilious or periodic fevers being known also, to arise from a smaller amount, or more diluted condition of the same circumstances, although more general and extensive, and more dependent on personal hygiene, it is in the power, as it is the duty of the civil authorities to mitigate, if they cannot entirely control them; and finally:

4th. That it was demonstrated, that by the proper application of curative measures, by the establishment of proper sanitary laws and police ordinances, rigidly enforced and effectually carried out, New Orleans can be made as healthy as any city in America; and that it was not only the interest of the city to accomplish these important purposes,—but that—

5th. A penalty could be as much enforced upon the civil authorities for neglecting the removal of conditions subversive of health and life, as for any purpose for which society was formed.

The Commission have spared no pains to do justice to the subject committed to their investigation. Circulars were sent to all persons whose opportunities of observation rendered it desirable to have their opinions on the *questiones vexatæ* connected with the epidemic.

The report, both in its manner and its matter, does credit to the Commission, and constitutes a valuable addition to our medical literature. J.

